# Why are we not screening for anal cancer routinely - HIV physicians’ perspectives on anal cancer and its screening in HIV-positive men who have sex with men: a qualitative study

**DOI:** 10.1186/s12889-015-1430-1

**Published:** 2015-01-31

**Authors:** Jason J Ong, Meredith Temple-Smith, Marcus Chen, Sandra Walker, Andrew Grulich, Jennifer Hoy, Christopher K Fairley

**Affiliations:** Melbourne School of Population and Global Health, University of Melbourne, Carlton, VIC 3010 Australia; General Practice and Primary Health Care Academic Centre, University of Melbourne, Carlton, VIC 3053 Australia; Melbourne Sexual Health Centre, Carlton, VIC 3053 Australia; Central Clinical School, Monash University, Clayton, VIC 3168 Australia; Kirby Institute, University of New South Wales, Darlinghurst, NSW 2010 Australia; Department of Infectious Diseases, Alfred Health and Monash University, 55 Commercial Road, Melbourne, VIC 3004 Australia

**Keywords:** Anal cancer, Screening, HIV, Men who have sex with men

## Abstract

**Background:**

Anal cancer is a priority health issue in HIV positive men who have sex with men. Anal cancer screening may be aimed at either detecting the precursor lesion (high grade anal intraepithelial neoplasia(HGAIN)) or early anal cancer. To date no qualitative study has explored the views of HIV physicians regarding anal cancer and its screening.

**Methods:**

We conducted indepth interviews with 20 HIV physicians (Infectious diseases, Immunology, Sexual health, General practice) in different settings (hospital, sexual health centres, general practice) from around Australia. Framework analysis was used to identify themes.

**Results:**

HIV physicians viewed anal cancer as a significant health issue and all agreed on the importance of anal cancer screening amongst HIV positive MSM if a valid screening method was available. Barriers for utilizing anal cytology was based primarily on the theme of insufficient evidence (e.g. no studies demonstrating reduction in mortality following screening or effective treatments for HGAIN). Barriers for utilizing DARE for early cancer detection were based on systemic factors (e.g. lack of opportunity, lack of priority, differences in HIV care practices); health provider factors (lack of evidence, difficulty discussing with patients, lack of confidence in DARE) and patient factors (perceived discomfort of DARE for patients, low anal cancer risk awareness). Physicians were willing to consider the idea of patient self-examination and partner-examination although concerns were raised regarding its reliability and issues surrounding partner dynamics.

**Conclusions:**

HIV physicians remain ambivalent regarding the most effective means to screen for anal cancer. More research is needed to address the physicians’ concerns before anal cancer screening can be implemented into routine HIV care.

## Background

Anal cancer occurs at higher rates in men who have sex with men(MSM) than in the general population [1] and with higher rates again in HIV-positive MSM [2]. Rates of anal cancer in HIV-positive MSM have been reported as high as 112 to 144 per 100,000 person years [3,4]. This is more common than other cancers such as colorectal cancer (73 per 100,000 person years) and lung cancer (55 per 100,000 person years) in the general population [5]. Anal cancer is now the most common non-AIDS-defining malignancy in those living with HIV [6].

Although anal cancer is seen to be important by HIV clinicians, few are actually screening for anal cancer [7]. There are several potential methods of screening being considered. Firstly, given the success with cervical cancer screening using cytology [8], anal cytology screening to detect the precursor lesion, high-grade anal intraepithelial neoplasia(HGAIN) has been proposed [9]. Secondly, some suggest that those at greatest risk for anal cancer (i.e. HIV-positive MSM) should be targeted with an annual digital ano-rectal examination(DARE) to detect early anal cancer [9-11]. If anal cancer is detected at an early stage, there are survival advantages with 5-year relative survival rates being 78% for localized anal cancer, 56% for regional disease and 18% for metastatic disease [12,13]. Thirdly, a novel means of early detection may be to encourage patients to detect early signs of cancer themselves or with the aid of their partner [11]. Although there is no research to support the efficacy of employing such methods, physicians often describe how patients would present because of an unusual lesion they have found themselves [14]. A further exploration of self- and partner-examination is warranted as they may become a useful adjunct to anal cancer screening.

To date, there are no published qualitative studies exploring the views of HIV physicians regarding anal cancer and its screening. We sought to gain an in-depth understanding of how key HIV clinicians view anal cancer and its screening using the methodologies described above.

## Methods

### Participants

Semi-structured in-depth telephone interviews were conducted with HIV physicians throughout Australia. Health care services for Australians living with HIV are provided in hospital based settings (Infectious Disease, Immunology specialists) or community based clinics (Sexual health physicians, high-HIV caseload General Practitioners(GP)) [15]. Purposive sampling (i.e. selective sampling that identifies particular characteristics of the study population) was used to maximize diversity of physicians. Selection was based on characteristics that the authors discussed and agreed upon that may potentially influence views on anal cancer screening - fulltime or part-time work, setting of HIV care, specialty and gender of the physician. We interviewed physicians until data saturation was reached (i.e. when additional interviews did not shed any further light on the issue).

### Procedure

Physicians from major HIV clinics in each state were approached via an email containing study participant information and consent form. If consenting, the interview was conducted over the phone(JO) using a pre-piloted semi-structured questionnaire. Structured questions captured physicians’ demographic data and the remainder of the interview contained open questions aimed at eliciting physicians’ understanding and attitudes towards anal cancer and its screening. Screening was defined as 1) using anal cytology and 2) early cancer detection using an annual DARE, self-examination and partner-examination. This study focused on anal cancer screening in HIV-positive MSM population.

### Analyses

Data were transcribed, organized electronically and assigned codes using NVIVO (QSR International Pty Ltd, version 10.0, 2012), a qualitative research software program. Data were analyzed using an iterative approach. After each interview a preliminary analysis was performed to allow the follow up of emerging issues to be included in subsequent interviews. Once all interviews were completed, content analysis was performed to group and label the data in order to identify emerging themes. Coding was conducted independently by two researchers(one sexual health clinician, one sexual health researcher) and then discussed with the research team to achieve consensus on common themes. Finally, coding for themes and concepts was used to frame the remaining data. We adhered to the qualitative research review guidelines (RATS) [16] in reporting the findings of this research.

This research was approved by the Alfred Health Human Ethics Committee (Project 31/13).

## Results

Of 22 physicians approached, two did not reply to the invitation email and one suggested another physician in their clinic. Demographics are summarized in Table 1. Illustrative quotes include gender, specialty and years in HIV practice of the participant (YiHP).

**Table 1:** **Demographics of HIV physicians**

	**Number of HIV physicians**
State	
- New South Wales	9
- Victoria	6
- Queensland	3
- Canberra	1
- Tasmania	1
Work hours	
- Full-time	11
- Part-time	9
Specialty	
- High HIV case-load General practitioner	7
- Sexual health physician	7
- Infectious disease physician or immunologist	6
Gender	
- Male	12
- Female	8

Mean age = 51.6 years (range 35–61).

Mean duration of working with HIV patients = 21.6 years (range 5–34).

Mean number of HIV patients seen per week = 19.4 men (range 1–50).

### Physicians’ understanding of anal cancer

Participants had an excellent grasp of causation, risk groups and rates of anal cancer. Almost all spoke of increasing age, human papillomavirus and smoking as important risk factors. Anal cancer was seen by all as a disease of concern.

…a devastating infection (F, Sexual health, 15 YiHP)

…potentially very nasty (M, General Practice, 28 YiHP)

…the last 25 years the rates have almost doubled I guess in Australia and similar patterns have been seen around the world (M, Infectious Disease, 15 YiHP)

Virtually all HIV physicians specifically identified HIV-positive MSM as the most at-risk group for anal cancer. However some (especially general practitioners) expressed discrepancy between reported rates and their own personal practice:

…I suspect the numbers have increased a bit over the last five or 10 years but they haven’t gone up like everyone was predicting where we were going to see an avalanche (M, General Practice, 28 YiHP).

Despite scepticism from some, the consensus from the group was that targeting screening for HIV-positive MSM was important. There was an underlying sense of urgency that something should be done.

…you’ve got a cancer that’s 100 times more common [in HIV-positive MSM compared with the general population] then we should be trying to do something about it (M, Infectious Disease, 27 YiHP)

### Views on anal cytology

The major theme expressed when discussing cytology screening for HGAIN was the lack of convincing evidence.

…still done on the basis of belief rather than evidence (M, Immunologist, 30 YiHP)

Issues of concern included the absence of evidence for screening using cytology in reducing morbidity and mortality from anal cancer; the high prevalence of AIN in HIV+ MSMs and thus the concern of subjecting large numbers of patients to unnecessary investigations; and the lack of availability for patients to have a high resolution anoscopy as follow up to an abnormal anal cytology. Participants noted the uncertainty around the natural history of AIN, the effectiveness of treatments and whether treating HGAIN would make a difference to anal cancer rates.

…just about all gay men with HIV will have abnormal cells and the natural history of those abnormal cells is still not understood enough really so we don’t know what to do once we’ve found those abnormal cells (M, General Practice, 22 YiHP)

Reflected in this reluctance to initiate anal cancer screening was a very strong theme calling for more evidence or guidelines before such screening should be introduced for HIV patients.

…one of the problems is that the current guidelines that we have in Australia don’t recommend any form of anal cancer screening (M, General Practice, 23 YiHP).

…screening tests require a much higher level of evidence than even treatment studies (M, Sexual Health, 34 YiHP)

### Views on DARE

Given the reluctance of HIV physicians in Australia to implement anal cancer screening using anal cytology, further interviewing enquired about the possibility of implementing an early cancer detection program instead. This was proposed as an annual visual inspection of peri-anal region together with a digital ano-rectal examination(DARE) by the HIV physician. It was interesting that the initial response from the majority (across all specialties and years of HIV experience) were positive.

…probably best practice (M, Immunologist, 30 YiHP)

…reasonable thing to do (F, Infectious Disease, 23 YiHP)

…correct means by which we should be screening… should be embraced (M, General Practice, 30 YiHP)

…sensible, cheap and easy thing to do (F, Immunologist, 5 YiHP),

…it’s going to pick up lumps that may well be early cancers and I guess that’s where the efforts should be made, really (M, General practice, 22 YiHP)

But despite the enthusiasm for DARE, the majority of HIV physicians admitted to not doing this routinely for their population of HIV positive MSM.

…I do it very rarely unless someone has symptoms (M, Infectious Disease, 5 YiHP)

When asked to elaborate why there was this discrepancy between the idea that DARE is a good one with the actuality of implementing DARE into routine care, multiple barriers acting at three levels were identified: systems, health provider and patient. Table 2 provides a summary of major themes under each of these headings.

**Table 2:** **Major themes of barriers for implementing DARE**

Systems factors	Lack of opportunity
	Unclear referral pathway
	Differences in HIV care practices
	No financial incentives
Health provider factors	Lack of evidence
	Difficulty discussing with patients
	Lack of confidence in DARE
Patient factors	DARE discomfort
	Low anal cancer risk awareness

1. *Systemic barriers*

A strong theme that emerged was the absence of a clinical routine to incorporate DARE. Thus ‘the main barrier is forgetting to do it’ (F, Sexual health, 12 YiHP). Physicians stated they had lost the opportunity to offer a DARE during checks for sexually transmitted infections as the option for self-collected anal swabs had increased in recent years.

…now we’re getting people to self-collect all of the specimens at sexual health screens which I think is terrible because it means that often nobody looks (M, General practice, 23 YiHP)

This was compounded by the need to manage the complexities and competing interests of multi-morbidity seen in an ageing HIV population. Many physicians talked about the difficulties in delivering what is recommended already in the guidelines(e.g. screening for cardiovascular complications) and it seemed that anal cancer was not always a priority.

…often when you're time poor and there’s other more pressing issues that the patient wants to discuss… [DARE] may be something that gets missed (M, Sexual health, 15 YiHP)

The frustration over lack of time was almost palpable in some, whilst not an issue for others:

…it’s just one more thing that needs to be done in too little amount of time (M, GP, 30 YiHP)

…it’s only once a year. Your average consultation now with a HIV patient is nowhere near as cluttered as it was 10 years ago… so it [DARE] wouldn’t be that big an intrusion. It would add a minute or two to the consultation (M, Sexual health, 34 YiHP)

Another frustration for some physicians was the lack of clarity around who should be conducting the screening and where to send patients presenting with symptoms of concern. It was noted that in Australia, HIV patients may be cared for by different specialists(Infectious Disease, Immunology, Sexual health physicians or General Practitioners(GP)) in various settings(hospital or sexual health centre or GP) and that some patients can be under the care of more than one specialist. A consequence of this can be the assumption that another doctor is doing the anal cancer screening.

…where does that responsibility - who does it lie with? I guess for some people they potentially would fall through the cracks in the system in that we’d say it was the GPs and the GP would say it was ours (F, Sexual health, 25 YiHP)

Some participants perceived that differences inherent in specialty training and care setting could be a barrier with the Infectious Diseases and Immunology trained physicians being less comfortable and their practice setting not readily set up for DARE.

…being a sexual health physician we don’t mind looking at bums at all… That could be quite different in a hospital outpatient environment for instance… ID clinics, as you know, have always avoided going anywhere near the genitalia (M, Sexual health, 34 YiHP)

One hospital physician suggested that GPs who effectively run small businesses are incentivized by payment systems for shorter consultations, which could act as a barrier.

…GPs won’t do it because there’s no money in it… if you’ve got to spend two or three minutes getting somebody undressed and doing an exam and then explaining their result then that’s time that the GPs won’t want to spend (M, Immunologist, 24 YiHP)

2) *Health provider barriers*

At the health provider level, a prominent theme was the current lack of published evidence for DARE primarily to do with its effectiveness in detecting early anal cancer and the cost-effectiveness of implementing this into routine HIV care.

…everybody would need to be convinced that the evidence was strong enough (M, Immunologist, 24 YiHP)

Beyond the need for evidence, a small number of physicians expressed difficulties in initiating the topic of anal cancer screening with patients.

…I must admit it’s not easy for me to bring that one up (F, General practice, 19 YiHP)

…feel awkward in raising the issue (M, Immunologist, 24 YiHP)

In terms of the procedure of DARE itself, it was predominantly the sexual health physicians who discussed doubts about their ability to pick up early cancers. But in general there was consensus by all participants regardless of their specialty, that they would benefit from more training in utilizing DARE for detection of anal cancer.

…given that it could be very small and you want to pick it up early otherwise you lose a lot of the benefit. So I don’t think it’s a very sensitive method. Also it’s so user dependent and I can’t see that I would feel confident in it (F, Sexual health, 15 YiHP)

Most of us learn it [rectal exams] on the wards as an intern for constipation and prostate checks… I think the actual checking for anal cancer is more detailed …and I don’t have a good handle on exactly what to feel for, what to worry about, what not to worry about, all that kind of thing (F, Immunologist, 5 YiHP)

3) *Patient barriers*

At the patient level, two main themes emerged. Firstly, physicians reported that there may be a level of patient discomfort in receiving a DARE. This may be due to perceived patient embarrassment, patients not feeling prepared for the examination and potential issues with past history of sexual assault or fear of disclosing sexual orientation.

…people don’t like to think and talk about it [anal cancer]… you have to wait until patients become symptomatic and not embarrassed to talk about it or show you before it can get diagnosed (F, Immunologist, 5 YiHP)

…A lot of patients feel it’s a bit of an invasion (M, General practice, 30 YiHP)

…there have been a few patients where they’ve said ‘oh look I haven’t prepared for that sort of examination’… the patient had some fears about not being clean (M, General practice, 29 YiHP)

Furthermore several physicians discussed that having a female physician perform the DARE may be hampered by patients’ view of regarding them as a maternal figure.

…there’s kind of a relationship builds up which is - it kind of almost an intimate friendship to some extent… almost it’s like being an aunt or an older sister… and you don’t let aunts and older sisters examine your backside comfortably (F, Infectious disease, 23 YiHP)

The second main theme revolved around the perception that health literacy concerning anal cancer risk remained poor in HIV positive MSM populations.

…I think the vast majority wouldn’t know that [anal cancer is] something that they’re particularly at risk of (M, General practice, 22 YiHP)

### Attitude towards early cancer detection using self- and partner-examination

We also explored other potential methods for early cancer detection. A majority of HIV physicians believed that self-examination was already occurring amongst HIV-positive MSM.

…often patients do present because they’ve felt a lump (M, General Practice, 29 YiHP)

…quite a lot of patients I’ve looked after are quite adept at examining their perianal region (F, Infectious Disease, 23 YiHP)

Some discussed the positive aspects of self-examination as patients becoming more educated, aware and involved in their own health.

…it definitely gives the patient some responsibility for their own health which I think is definitely worth it (F, Sexual Health, 15 YiHP)

…it’s probably a bit like breast cancer screening… that if they find a lump that’s a good thing if they tell us about that before we find it in them (M, Infectious Disease, 5 YiHP)

However, it was not seen as a screening method that could be solely relied upon as there were issues regarding potential difficulties of the technique of performing a self-examination and the ad hoc nature it was being done.

…I don’t think the majority would to do it regularly and actually report back findings… There’s been campaigns to get men to do testicular self-examination. By and large not even that happens (M, Immunologist, 30 YiHP)

…[The anus is] not the easiest place to self-examine (M, Sexual Health, 22 YiHP)

…they don’t know what they’re feeling and they haven’t had the chance to be trained (M, Immunologist, 24 YiHP)

There was also a common perception that partner-examination was already happening.

…plenty of boys do, as part of foreplay, stick their fingers in each other’s butts and are probably fairly familiar with what they feel (M, General Practice, 30 YiHP)

Although it was seen as easier to do in comparison to self-examination, it was perceived that less patients would be willing to ask their partner to examine their anus for a medical reason. Concern was expressed over the changing role of the partner:

…putting the responsibility on to the partner for the screening. I don’t think that’s appropriate’ (F, Sexual Health, 14 YiHP).

It was noted that there may be a blurring of the line between sexual pleasure and examination for abnormalities, partnerships may not last and partners who are not trained don’t understand what to feel for. Physicians stated that the partner finding unusual lesions may increase anxiety unnecessarily for both the patient and their partner. There was also a risk that if the physician was out of the loop for screening that significant lesions may be missed.

…it’s not very romantic to have your partner do it too often I think. Well you’d need a very good friend at least’ (M, Sexual Health, 22 YiHP)

## Discussion

This study is the first to report qualitative data from HIV physicians on current views about anal cancer and attitudes regarding anal cancer screening in HIV-positive MSM. It is clear that there is excellent awareness of the issue of anal cancer amongst HIV physicians from all specialties with varying years of experience. Consistent with quantitative studies, there was an urgency that action is needed [9-11].

Regarding anal cytology, there were two major barriers highlighted by participants. Logistically, there is a mismatch between potentially high number of men with an abnormal result from screening and the paucity of physicians able to perform high resolution anoscopy to follow up these abnormalities. Currently in Australia, only a handful of physicians are trained in providing high resolution anoscopy and virtually all are conducted within a research setting. Secondly, there was a call for greater level of evidence to demonstrate that treatment of HGAIN reduces the incidence of anal cancer and the effectiveness of treatments of HGAIN to resolve these lesions. Until more high resolution anoscopists are trained, and anal cytology is reflected in HIV guidelines supported by a higher level of evidence than ‘expert opinion’, it seems unlikely that Australian physicians would utilize anal cytology as a means of screening.

Whilst the majority of literature is focused on anal cancer screening using cytology, this study provided detailed views of HIV physicians in implementing an early cancer detection model using an annual DARE. DARE is regarded as the principal cancer screening test compared with anal cytology, which is the principal precursor (HGAIN) screening test [9]. Some researchers consider that DARE should be performed in everyone who is at high risk of anal cancer [11]. However, these recommendations have not been widely reflected in regional or national HIV guidelines [17]. Although many physicians were supportive of early detection of anal cancer, they identified multiple potential barriers at the systems, provider and patient levels. This highlights that even though an annual DARE appears to be a relatively simple procedure, it may not necessarily be easy to implement.

At the systems level, participants discussed the need to set in place a clear system that is clinic-specific that makes DARE a routine part of HIV care. There may be value in utilizing the knowledge gained from system change literature [18] to mitigate barriers to implementation. For instance by having a clear system in place, a Canadian HIV clinic increased its anal cancer screening either by DARE and/or cytology from 10% to 44% of patients over 3 years [19]. Furthermore, the provision of incentives should be explored in different settings if DARE becomes widely recommended as a routine part of HIV care as providing incentives has been found to be important for increasing uptake of other screening tests such as pap tests and mammograms [20].

Our study suggests that provider level factors must be addressed. A key barrier was even though DARE is recommended as a means of early detection of anal cancer in some guidelines [21,22], the level of evidence provided is only by ‘expert opinion’. Almost all physicians remained skeptical over incorporating DARE into routine care until stronger evidence was made available. To date, there has been no published data on the sensitivity and specificity for DARE to detect early anal cancer. However, anal cancers were detected with an average size of 2.9 cm at diagnosis in HIV-positive patients, and most were visible and/or palpable for some time before definitive diagnosis [23]. Although it is likely that regular DARE could improve detection of early anal cancer, no studies have been published to evaluate whether it is cost-effective and would lead to a reduction in morbidity and mortality from anal cancer. As physicians in this study highlighted, these data must be shown before DARE becomes widely adopted. Although participants were comfortable with performing a rectal examination for prostate cancer screening, using DARE for anal cancer screening was seen as a new skill that required extra training. Instead of bypassing the anal canal to examine the prostate, physicians now have to learn to feel the entirety of the anal canal and identify the presence of any lumps or ulcers that may potentially be anal cancer.

Physicians had a perception that patients may find DARE uncomfortable. This was not surprising and was consistent with other screening programs involving genital examination – cervical pap smears [24] and mammogram [25]. Physicians involved in an anal cancer study also expressed concern that some patients may worry about being clean for the examination [26]. Therefore, one strategy to increase the uptake of DARE may be to allow time for patients to prepare psychologically and physically for the examination. This may involve telling patients that a DARE is due at their next visit or give them the option to clean themselves before doing the DARE.

Self-examination and partner-examination are potentially novel ways of screening for anal cancer. Whilst there are data for self-examination for breast cancer [27], skin cancer [28], and testicular cancer [29], this is the first published study exploring the views of HIV physicians regarding self- and partner-examination as modes for early anal cancer detection. Physicians were open to discuss the concept of these novel screening methods and if the issues raised in this paper can be addressed, they may prove to be a useful adjunct to anal cytology and/or DARE. For example, in addition to regular screening by a physician, patients may be educated in how to self-examine and/or recognize potential symptoms of anal cancer and be encouraged to present to the physician between their regular scheduled physician screening. However, as with DARE, more research is needed to test its efficacy and acceptability before this is recommended to patients.

Despite multiple barriers identified in this study, these are not insurmountable and can be addressed. For instance, at the systems level, having a clear reminder system, a clinic champion (to encourage screening) and clear referral pathways may help to facilitate more men being screened. At the health provider level, the provision of more research evidence and training may help physicians feel more confident in conducting screening. And at the patient level, strategies to improve their awareness of anal cancer risk and allowing adequate preparation time before screening may facilitate greater uptake of screening.

In common with all qualitative studies, the findings are context dependent. However the inclusion of HIV physicians with different backgrounds and work settings across Australia has offered a broad range of viewpoints. This study has provided detailed information about current issues that need to be addressed before anal cancer screening be implemented. It may be useful to explore how widely these views are held in a quantitative study of a larger number of HIV physicians. The other limitation pertains to the current Australian practice of not offering anal cancer screening outside a research setting. This meant that the vast majority of physicians interviewed were not participating in any anal cancer screening and thus influenced the tone of the study with multiple barriers identified. Future research to specifically include physicians from overseas who are actively screening may provide insights into facilitators of anal cancer screening.

## Conclusion

The best method for anal cancer screening is still an area of uncertainty for HIV physicians in Australia. More evidence (e.g. of reduction in mortality and morbidity of anal cancer as a result of screening) may be needed for anal cytology and DARE before it is implemented. Inclusion of an annual DARE as targeted screening for HIV-positive MSM may not be easy to implement with multiple barriers needing to be addressed. Consideration may be given to self-examination and partner-examination as adjuncts to screening if issues of its reliability could be addressed and if partners would feel comfortable and competent to take on this role.

## Abbreviations

DARE: Digital ano-rectal examination
GP: General practice/general practitioner
HGAIN: High grade intra-epithelial neoplasia
MSM: Men who have sex with men
